# Glycogen synthase AfGsy2 regulates mycelial growth, stress response, predatory activity, and chlamydospore development in the nematode-trapping fungus *Arthrobotrys flagrans*

**DOI:** 10.3389/fcimb.2026.1891167

**Published:** 2026-10-07

**Authors:** Qianfei Shi, Yu Zhang, Minghe Mo, Guohong Li

**Affiliations:** 1 State Key Laboratory for Conservation and Utilization of Bio-Resources in Yunnan, Yunnan Key Laboratory of Basic Research and Innovative Application for Green Biological Production, Yunnan University, Kunming, China; 2 School of Ecology and Environment, Yunnan University, Kunming, China

**Keywords:** glycogen metabolism, nematophagous fungi, pathogenicity, sporulation, trap morphogenesis

## Abstract

**Introduction:**

Glycogen is a universal carbon and energy reserve that plays crucial roles in fungal stress adaptation, cellular homeostasis, and predatory activity. However, the role of glycogen metabolism in nematode-trapping fungi has largely remained unexplored. Here, we performed a systematic functional characterization of *AfGsy2*, the glycogen synthase-encoding gene in the biocontrol fungus *Arthrobotrys flagrans*.

**Methods:**

Using a CRISPR-Cas9-mediated deletion mutant, together with overexpression and complementation strains, we investigated the involvement of AfGsy2 in mycelial growth, stress tolerance, trap formation, nematicidal activity against *Caenorhabditis elegans*, chlamydospore production and germination, and glycogen accumulation. Subcellular localization was examined using an AfGsy2-GFP fusion protein.

**Results:**

Deletion of *AfGsy2* reduced hyphal growth on both nutrient-rich and nutrient-poor media but enhanced tolerance to osmotic, salt, and oxidative stresses, suggesting that blocked glycogen synthesis may trigger metabolic rerouting toward alternative stress-protective compounds. Notably, the *ΔAfGsy2* mutants exhibited reduced trap production and nematicidal activity against *C. elegans*, whereas overexpression of *AfGsy2* accelerated these processes, suggesting a link between glycogen metabolism and predatory activity. Moreover, AfGsy2 positively regulated chlamydospore production, glycogen accumulation, and chlamydospore germination. AfGsy2-GFP predominantly localized to the vacuoles of chlamydospores, and complementation restored the mutant phenotypes to wild-type levels.

**Discussion:**

Collectively, these findings uncover a link between glycogen metabolism and growth, stress resilience, sporulation, and predatory activity in *A. flagrans*, and establish a molecular foundation for the rational improvement of nematode-trapping fungi. Together, these results identify AfGsy2 as a pleiotropic regulator linking glycogen metabolism to biocontrol-related traits.

## 1 Introduction

In fungi, glycogen serves as a primary carbon and energy reserve, enabling cells to withstand carbon scarcity by serving as a stored carbon source that can be mobilized when needed (Gilbert et al., 2010; Wilson et al., 2010). Fungal glycogen synthesis relies on the concerted action of glycogen synthase (Gsy) and glycogen branching enzyme (Gbe), whereas degradation is mediated by glycogen phosphorylase (Gph). Together, these three enzymes constitute a dynamic metabolic network that responds to fluctuations in carbon availability (Fontana and Krisman, 1978; Thines et al., 2000; Zhu et al., 2025). In model yeasts and pathogenic fungi, glycogen metabolism has been shown to influence stress adaptation, cell wall integrity, and virulence (Wu et al., 2001; Loza and Doering, 2024; Miao et al., 2024), underscoring its regulatory functions beyond energy storage.

Glycogen metabolism is also intimately linked to cell wall integrity and virulence in plant-pathogenic fungi. For example, in *Magnaporthe oryzae*, disruption of glycogen degradation impairs appressorium function and pathogenicity, whereas blocking glycogen synthesis alone does not affect virulence (Badaruddin et al., 2013). Similarly, in *Fusarium oxysporum*, glycogen catabolism influences pathogenicity toward plant hosts (Corral-Ramos and Roncero, 2015), and in *Cryptococcus neoformans*, glycogen synthesis is coupled to cell wall glucan organization (Loza and Doering, 2024). Collectively, these studies demonstrate that glycogen metabolism is not merely an energy reservoir but also participates broadly in stress adaptation, cell wall assembly, and pathogenicity via the coordinated regulation of its synthesis, mobilization, and spatial organization.

However, existing studies have largely focused on model yeasts and plant-pathogenic fungi (Walkey et al., 2011; Badaruddin et al., 2013), whereas the role of glycogen metabolism in nematode-trapping fungi remains poorly understood. These fungi capture nematodes via specialized trapping devices and represent promising sources of biological control agents. *Arthrobotrys flagrans* (formerly *Duddingtonia flagrans*) forms adhesive three-dimensional networks to trap nematodes and produces chlamydospores, traits that make it an attractive candidate for biocontrol development (Monteiro et al., 2018; Yu et al., 2021; Balbino et al., 2022). Indeed, chlamydospore-based formulations (e.g., Bioverm^®^) have been successfully applied to control nematode infections in livestock (Céspedes-Gutiérrez et al., 2021; Mendes et al., 2023). Notably, *A. flagrans* has also been reported to exhibit trapping activity against various nematode species, including some plant-parasitic nematodes (Monteiro et al., 2020; Wernet and Fischer, 2023), pointing to its broader nematicidal potential. However, the molecular mechanisms governing these biocontrol-related traits, particularly the contribution of glycogen metabolism, remain largely elusive. Therefore, systematic functional characterization of AfGsy2, the glycogen synthase of *A. flagrans*, and elucidation of its roles in growth, chlamydospore formation, and predatory activity, would significantly advance our understanding of carbon metabolic regulation in nematode-trapping fungi and provide a mechanistic foundation for the rational improvement of biocontrol properties. Our whole-genome sequencing and transcriptomic data revealed that the *A. flagrans* genome harbors only a single Gsy2 homologue. This unique feature makes the gene an ideal target for dissecting the link between glycogen metabolism and biocontrol-related traits, as its disruption or overexpression is expected to yield more direct phenotypic consequences. Collectively, this work provides a foundation for understanding the biological functions of glycogen metabolism in nematode-trapping fungi through systematic characterization of AfGsy2 in regulating growth, sporulation, and predatory activity.

## 2 Materials and methods

### 2.1 Strains and culture conditions

*Arthrobotrys flagrans* CBS 565.50 and *Caenorhabditis elegans* were preserved in the Microbial Library of the Germplasm Bank of Wild Species from Southwest China. The wild-type (WT) strain of *A. flagrans* was inoculated onto potato dextrose agar (PDA) medium and incubated at 28 °C. The gene deletion strains (Δ*AfGsy2*) were cultured on PDA medium supplemented with 100 μg/mL hygromycin B (Sigma-Aldrich, 400051), and the gene overexpression and complementation strains (OE*AfGsy2*, Δ*AfGsy2^C^*) were cultured on PDA medium supplemented with 50 μg/mL G418 (Sigma-Aldrich, G8168) (**Supplementary Table 1**). *C. elegans* was grown on nematode growth medium (NGM) plates seeded with *Escherichia coli* OP50 as a food source.

### 2.2 Gene structure, domain architecture, and phylogenetic analysis

In this study, the *AfGsy2* gene sequence was obtained from the *A. flagrans* genome dataset (https://www.ncbi.nlm.nih.gov/datasets/genome/GCA_030406815.1/). The gene structure of *AfGsy2* was analyzed using the Gene Structure Display Server (http://gsds.cbi.pku.edu.cn/). Conserved protein domains were identified using the NCBI CD-Search tool (https://www.ncbi.nlm.nih.gov/Structure/bwrpsb/bwrpsb.cgi). Phylogenetic tree construction was performed in MEGA X using the maximum likelihood method, with 1,000 bootstrap replicates to provide branch support.

### 2.3 Genomic DNA extraction from *A. flagrans*

*Arthrobotrys flagrans* was inoculated onto PDA medium and incubated at 28 °C for 4–5 days. Mycelia were harvested using a sterile spatula, snap-frozen in liquid nitrogen, and thoroughly ground in a mortar. Subsequently, genomic DNA was extracted using the HiPure Fungal DNA Mini Kit III (Magen, D3175-02).

### 2.4 Construction of *AfGsy2* gene deletion, overexpression, and complementation vectors

In this study, CRISPR-Cas9-mediated homologous recombination was employed to disrupt the *AfGsy2* gene. First, based on the whole-genome sequence of *A. flagrans*, sgRNA was designed using CRISPOR software (https://crispor.gi.ucsc.edu/crispor.py) (Concordet and Haeussler, 2018). The sgRNA target sequence was predicted to be unique in the genome based on *in silico* analysis (Zhang et al., 2023b), with no high-similarity off-target sites identified by sequence alignment. Next, the homology arms were amplified from genomic DNA, and the hygromycin resistance gene (*hph*) was amplified from the pCSN44 plasmid. The three fragments (upstream arm, *hph*, and downstream arm) were then directionally assembled into the pCE-Zero vector using the ClonExpress Ultra One-Step Cloning Kit (Vazyme, C115-02). Finally, Cas9 protein (20 μM), Cas9 NLS Reaction Buffer, and chemically synthesized sgRNA (10 μM) were mixed at a ratio of 1:1:8 to form the Cas9-sgRNA ribonucleoprotein (RNP) complex *in vitro*. The sgRNAs and primers used for fragment amplification in this study were synthesized by Tsingke Biotechnology Co., Ltd. (**Supplementary Table 2**).

Following a previously described method (Zhang et al., 2025), we used the *H2B* promoter to drive *AfGsy2* overexpression and to visualize the subcellular localization of the AfGsy2–GFP fusion protein. The entire expression cassette was targeted to a site 0.5 kb downstream of the *ku70* gene. Briefly, the *H2B* promoter (2.0 kb), the *AfGsy2* gene sequence, EGFP, the *H2B* terminator (1.0 kb), and a G418 resistance cassette were sequentially assembled into pUC19 vector. To enable site-specific integration, 1.0-kb homology arms flanking the insertion site were placed upstream of the *H2B* promoter and downstream of the G418 resistance cassette, respectively. To exclude potential effects of the GFP tag on protein function and localization, we generated an untagged OE*AfGsy2* strain (n-AfGsy2) and a GFP-only strain; the former served as a functional control, and the latter as a fluorescence control.

The complementation vector was constructed similarly, with the same integration target (0.5 kb downstream of *ku70*). This vector contained, in sequential order, the *ku70* upstream homology arm, the full-length *AfGsy2* gene under its native promoter and terminator, EGFP, a G418 resistance cassette, and the *ku70* downstream homology arm. Primers (**Supplementary Table 2**) for amplifying each fragment were designed using CE Design software. All fragments were assembled into pUC19 vector with the ClonExpress Ultra One-Step Cloning Kit (Vazyme, C115-02).

### 2.5 Protoplast preparation of *A. flagrans*

To generate the *AfGsy2* gene deletion and overexpression mutants, the corresponding cassettes were introduced into the wild-type (WT) strain, whereas the complementation cassette was introduced into the Δ*AfGsy2* mutant strains. Briefly, the WT strain was cultured on PDA, and the Δ*AfGsy2* strain on PDA containing hygromycin B (100 μg/mL), at 28 °C for 3–4 days. Mycelial plugs were then transferred to PDB and incubated at 28 °C with shaking at 180 rpm for 24 h. Mycelia were collected by filtration through sterile filter paper, washed with STC buffer (1 M sorbitol, 50 mM CaCl_2_, 10 mM Tris-HCl), and resuspended in 15–20 mL cell wall digestion solution. Digestion was carried out at 28 °C with gentle agitation (90 rpm) for 4–5 h. The resulting protoplasts were filtered, pelleted by centrifugation (4 °C, 3,000 × *g*, 6 min), and then mixed with 6–10 μg of cassettes per 10^7^ protoplasts. After a 30-min incubation on ice, 1 mL PTC buffer (10 mM Tris-HCl [pH 7.5], 50 mM CaCl_2_, 50% [wt/vol] PEG 3350) was added, gently mixed by pipetting, and the mixture was incubated at 28 °C for 40 min. The transformation mix was spread onto PDASS regeneration medium (PDA supplemented with 0.6 M sucrose, 0.3 g/L peptone, 0.3 g/L tryptone, 0.3 g/L yeast extract, and 8 g/L agar) in 9 cm Petri dishes. Plates were sealed and incubated at 28 °C for 48 h. Transformants were selected on PDA supplemented with either hygromycin B (100 μg/mL) or G418 (50 μg/mL), as appropriate.

### 2.6 Screening and verification of mutant strains

Mycelia (~1.0 g) from the WT, Δ*AfGsy2*, OE*AfGsy2*, and Δ*AfGsy2^C^* strains were harvested from the plates using a sterile spatula, mixed with quartz sand and urea extraction buffer (420.42 g/L urea, 6.05 g/L Tris, 3.65 g/L NaCl, 1% SDS, pH 8.0), and homogenized in a high-throughput tissue homogenizer. Subsequently, genomic DNA was extracted using the HiPure Fungal DNA Mini Kit III (Magen, D3175-02). To verify gene deletion in the Δ*AfGsy2* strains, a 1.0 kb region spanning the sgRNA target site (0.5 kb upstream and 0.5 kb downstream) was amplified by PCR using gene-specific primers F and R (**Supplementary Table 2**). For the OE*AfGsy2* and Δ*AfGsy2^C^* strains, correct integration of the expression cassettes at the targeted locus (0.5 kb downstream of the ku70 gene) was confirmed by PCR using primers flanking the integration site (**Supplementary Table 2**). In addition, transcript levels of *AfGsy2* in the OE*AfGsy2* and Δ*AfGsy2^C^* strains were examined by RT-qPCR to confirm overexpression and restoration of expression, respectively.

### 2.7 Mycelial growth rate analysis

The WT strain was cultured on PDA, the Δ*AfGsy2* mutant on PDA supplemented with hygromycin B (100 μg/mL), and the OE*AfGsy2* and Δ*AfGsy2^C^* strains on PDA supplemented with G418 (50 μg/mL) at 28 °C for 4–5 days. Mycelial plugs (0.8 cm in diameter) taken from the colony margins were inoculated onto PDA, tryptone glucose agar (TG, glucose 10 g/L, tryptone 10 g/L, agar 15 g/L), and tryptone yeast extract glucose agar (TYGA, glucose 10 g/L, tryptone 10 g/L, yeast extract 5 g/L, molasses 5 g/L, agar 15 g/L) plates and incubated at 28 °C. Colony diameters were measured daily for five consecutive days, and photographs were taken. Three independent experiments were performed, and growth curves were plotted using GraphPad Prism 10 (San Diego, CA, USA).

### 2.8 Chemical stress tolerance assays

Chemical stress tolerance was evaluated as described previously (Zhang et al., 2023b). Mycelial plugs (0.8 cm) of each strain were placed on PDA plates supplemented with sorbitol (0.25, 0.5, 1 M), SDS (0.01%, 0.02%, 0.03%), Congo Red (25, 50, 100 μg/mL), NaCl (0.1, 0.2, 0.3 M), or H_2_O_2_ (2.5, 5 mM). Plates were incubated at 28 °C for 5 days, and colony diameters were measured daily. The relative growth inhibition (RGI) values were calculated using the formula: RGI (%) = [(control colony diameter − treated colony diameter)/control colony diameter] × 100%. Three biological replicates were performed for each condition.

### 2.9 Trap formation and nematicidal activity assays

Mycelial plugs of each strain were cultured in 250 mL potato dextrose broth (PDB) at 28 °C with shaking at 180 rpm for 12 h. Mycelia were harvested by filtration through a sterile funnel, washed extensively with sterile water until the filtrate became colorless, and resuspended in 5 mL sterile water. The suspension was evenly spread onto 3.5 cm water agar (WA) plates and incubated at 28 °C for 2–3 days to allow hyphal recovery. Synchronized L3-stage *C. elegans* larvae were washed, and approximately 200 nematodes were added to each plate. Plates were then incubated at 28 °C to induce trap formation.

Trap formation was quantified directly on WA plates under a light microscope. Each plate was evenly divided into eight equal-area sectors, and three sectors were randomly selected for trap counting. The average of the three sectors was multiplied by 8 to estimate the total number of traps per plate, then divided by the plate area to calculate the number of traps per cm². For each strain, three independent biological replicates were performed. Within each biological replicate, three technical replicate plates were counted, and the mean of the three technical replicate plates was used as the biological replicate value.

For nematode mortality assays, nematodes were assessed at 12, 24, and 36 h post-inoculation, with nematodes counted across the entire plate under a light microscope. Nematodes were scored as dead when they failed to respond to gentle probing with a fine needle and showed no visible movement. Three biological replicates were performed per strain.

### 2.10 Chlamydospore morphology observation, quantification, and germination rate assay

Mycelial plugs of each strain were cultured in 250 mL PDB (28 °C, 180 rpm) for 12 h. Mycelia were collected, washed, and resuspended in 5 mL sterile water as described above. The suspension was spread onto 6 cm WA plates (supplemented with 2.5 g/L glucose) and incubated at 28 °C. Chlamydospore formation was monitored daily. On day 14, each plate was evenly divided into eight equal-area sectors, and three sectors were randomly selected for chlamydospore counting under a light microscope. The average count from the three sectors was multiplied by 8 to estimate the total number of chlamydospores per plate, which was then divided by the plate area to calculate the number of chlamydospores per cm². For each strain, three independent biological replicates were performed. Within each biological replicate, three technical replicate plates were counted, and the mean of the three technical replicate plates was used as the biological replicate value.

Chlamydospores used for germination assays were prepared as follows. Sterile water and glass beads were added to 14-day-old WA plates, and the plates were shaken to release spores. The chlamydospores suspension was collected and centrifuged at 3,000 rpm for 5 min, and the supernatant was discarded. The pellet was washed twice with sterile water and resuspended in 1 mL sterile water, followed by thorough mixing. A 30 μL aliquot was placed on a glass slide, covered with a coverslip, and counted directly under a light microscope. Based on the counts, the spore concentration was adjusted with sterile water to approximately 250 chlamydospores per 100 μL, and a 100 μL aliquot was spread onto freshly prepared 6 cm WA plates. Germination was scored under a light microscope at 3, 6, 12, and 24 h. At least 200 spores were assessed per biological replicate. A spore was considered germinated when the germ tube length exceeded half the spore diameter. Three biological replicates were performed.

### 2.11 Glycogen staining and subcellular localization observation

Glycogen accumulation was visualized by Lugol’s iodine staining, which reflects the relative glycogen level based on staining intensity. Mycelia and chlamydospores at early, middle, and late developmental stages were sampled and stained with 50 μL Lugol’s iodine solution (Sigma-Aldrich, 62650) for 30 s, after which excess iodine was removed using absorbent paper. Glycogen-associated staining intensity was observed and documented under a light microscope (Youssar et al., 2019). Three biological replicates were performed for each strain.

Mycelial plugs of the WT, OE*AfGsy2*, and *gfp* control strains were inoculated into 250 mL PDB and cultured at 28 °C with shaking at 180 rpm for 12 h. Mycelia were harvested, washed, and resuspended in 5 mL sterile water, then spread onto 6 cm WA plates (supplemented with 2.5 g/L glucose) and incubated at 28 °C for 24 h. Subsequently, a small amount of mycelium was scraped onto a glass slide, and 80–100 μL of CMAC staining solution (7-amino-4-chloromethylcoumarin; Thermo Fisher Scientific, C2110) at a final concentration of 100 μM was added. The samples were stained at room temperature for 40 min in the dark, washed 3–5 times with sterile water, and observed under a confocal laser scanning microscope (excitation/emission: 353/466 nm). At least 10 chlamydospores were examined per biological replicate, with three biological replicates performed.

### 2.12 RNA extraction and real-time quantitative PCR analysis

The WT, Δ*AfGsy2*,OE*AfGsy2*, and Δ*AfGsy2^C^* strains were cultured on PDA at 28 °C for 3–4 days. Mycelia (~0.5 g) were harvested using a sterile pipette tip. Total RNA was extracted using the UNIQ-10 column Trizol total RNA extraction kit (Sangon Biotech, B511321-0100) and reverse-transcribed into cDNA with the HiScript III 1st Strand cDNA Synthesis Kit (Vazyme, R312-01). RT-qPCR was performed on a Roche LightCycler 480 system (Roche Applied Science) using ChamQ SYBR qPCR Master Mix (Vazyme, Q311-02) and gene-specific primers (**Supplementary Table 2**). The *gpd* gene served as the internal reference. Relative transcript levels were calculated using the 2^–ΔΔCT^ method based on three biological replicates.

### 2.13 Prediction and structural assessment of protein–protein interactions

Candidate AfGsy2-interacting proteins were identified using the STRING database (v12.0) against the *A. flagrans* proteome, with a minimum required interaction score set at high confidence (0.700). Functional annotations of candidate interactors were retrieved directly from STRING. To evaluate the plausibility of direct physical association, the binary complex between AfGsy2 and each candidate was predicted using AlphaFold3 (https://alphafoldserver.com/) with default parameters. Overall model confidence was assessed by the predicted TM-score (pTM), and interface confidence was quantified by the interface predicted TM-score (ipTM). Following established guidelines, ipTM > 0.75 was considered indicative of a highly probable direct interaction, 0.5–0.75 of moderate confidence, and < 0.5 of a low likelihood for a stable physical complex.

### 2.14 Transcriptomic data source and analysis

The RNA-seq data analyzed here were obtained from our previously published study (Zhang et al., 2026). These data represent the transcriptome of wild-type *A. flagrans* during chlamydospore formation. In brief, *A. flagrans* was cultured on WA plates (supplemented with 2.5 g/L glucose) to induce chlamydospore formation. The culture conditions and induction of chlamydospores were performed following the same procedures described in Sections 2.10 and 2.11. Samples were collected at 0, 1, and 3 days after induction on WA plates, corresponding to the hyphal stage and early-to-late chlamydospore developmental stages, with three biological replicates per time point. Total RNA was extracted, sequencing libraries were prepared and paired-end sequencing was performed on an Illumina platform. Reads were filtered by fastp, aligned with HISAT2, and differentially expressed genes were identified using |log_2_(fold change)| ≥ 2 and *p* < 0.05.

### 2.15 Data analysis

For each assay, at least three independent biological replicates were performed. Within each biological replicate, technical measurements were averaged to obtain a single value for that replicate. All quantitative data are presented as mean ± SD calculated from these biological replicates (n = 3), and all statistical tests were performed on these biological replicate means. Statistical analyses were performed using GraphPad Prism (version 10). Normality and homogeneity of variances were verified using the Shapiro-Wilk test and Bartlett’s test, respectively. For comparisons involving multiple strains across different concentrations, media, or time points, data were subjected to one-way or two-way analysis of variance (ANOVA), followed by Tukey’s honest significant difference (HSD) *post-hoc* test for multiple comparisons. For time-course experiments (including mycelial growth, trap formation, nematode mortality, and chlamydospore germination), two-way ANOVA with strain and time as independent variables was applied, followed by Tukey’s multiple comparison test. Exact *p*-values are reported in the corresponding figure legends, with statistical significance defined as: *p < 0.05; **p < 0.01; ***p < 0.001; ****p < 0.0001.

## 3 Results

### 3.1 Gene structure and protein sequence analysis of AfGsy2

The *AfGsy2* gene contains a 2,049 bp coding sequence comprising five exons and encodes a protein of 702 amino acids with a predicted molecular mass of 79.5 kDa. AfGsy2 homologs with known biological functions from other fungi were selected, and their GenBank accession numbers are indicated in **Figure 1**. Conserved domains were visualized using NCBI Batch CD-Search and TBtools, and a phylogenetic tree was constructed using MEGA X with the maximum likelihood method. As shown in **Figure 1**, AfGsy2 from *A. flagrans* clustered closely with homologs from the filamentous fungi *Dactylellina haptotyla*, *Drechslerella dactyloides*, *Pyricularia oryzae*, *Fusarium graminearum*, and *F. oxysporum*, whereas it was phylogenetically distant from those of the unicellular fungi *Cryptococcus neoformans* and *C. albicans* (**Figure 1**). Conserved domain analysis revealed that the GSY homologs from all seven examined species harbor a GT3_GSY2-like domain and an RfaB domain (**Figure 1**), indicating that both domains are broadly conserved across fungi. The RfaB domain is a conserved glycosyltransferase (GT) domain that participates in lipopolysaccharide (LPS) core oligosaccharide synthesis in bacteria, functioning as a key enzyme in Gram-negative bacterial outer membrane biogenesis, and belongs to the GT-B glycosyltransferase superfamily (Peters et al., 2020).

**Figure 1:**
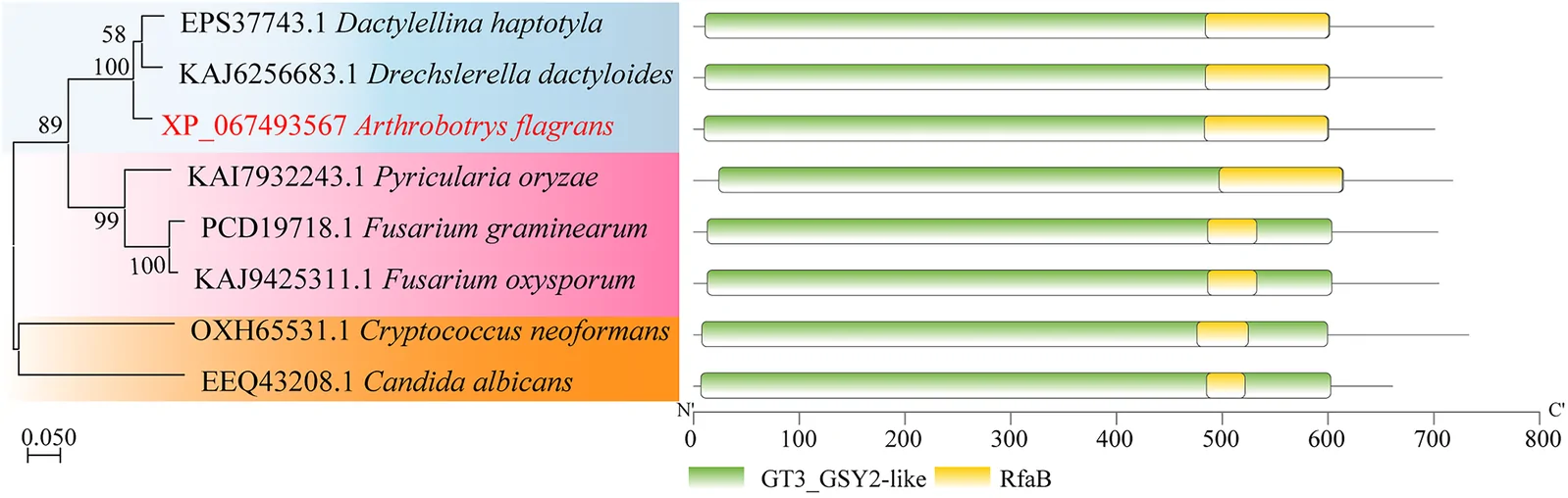
Maximum likelihood phylogenetic tree and conserved domains based on the amino acid sequences of Gsy2 homologous proteins from different fungi.

### 3.2 Construction and verification of AfGsy2 deletion, overexpression, and complementation strains

To investigate the biological function of AfGsy2, we generated *AfGsy2* gene deletion mutants (Δ*AfGsy2*) via CRISPR-Cas9-mediated homologous recombination, yielding 20 knockout transformants that displayed consistent phenotypes. Agarose gel electrophoresis confirmed successful integration of the hygromycin-resistance cassette at the target locus, as evidenced by the amplification of an approximately 3.1 kb fragment in the Δ*AfGsy2* mutants, compared with a 1.0 kb fragment in the wild-type strain (**Figures 2A–C**). Similarly, the overexpression and complementation cassettes were integrated at a site 0.5 kb downstream of the *ku70* gene to generate the OE*AfGsy2* and Δ*AfGsy2^C^* strains, respectively (**Figure 2D**). RT-qPCR analysis revealed no detectable AfGsy2 transcript in the Δ*AfGsy2* strains, whereas expression levels in the Δ*AfGsy2^C^* strains were restored to levels comparable to that of the WT. In the OE*AfGsy2* strains, AfGsy2 transcript levels were significantly elevated, reaching 5- to 5.5-fold of the WT level (**Figure 2E**).

**Figure 2:**
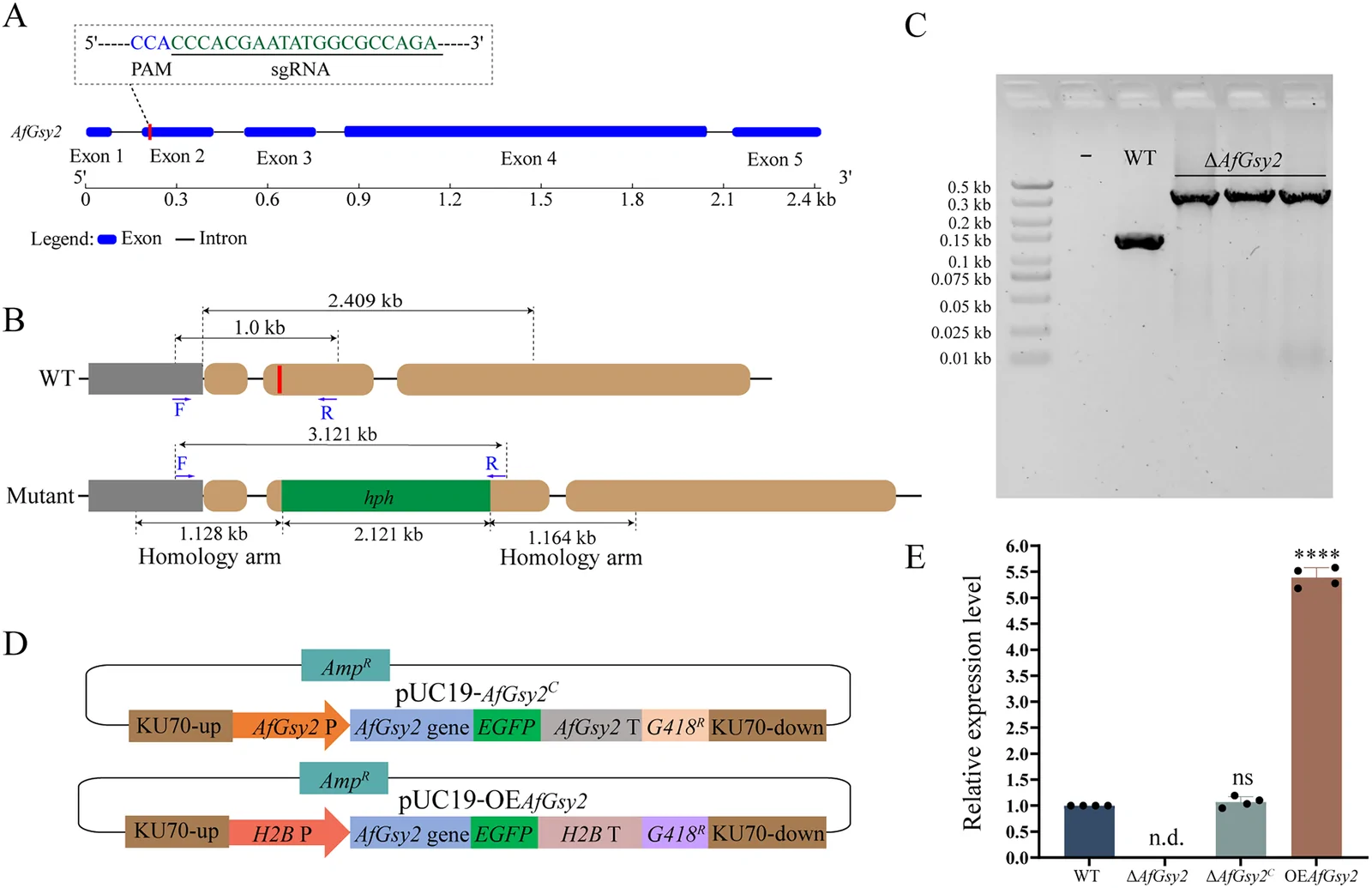
Construction and verification of *AfGsy2* deletion, overexpression, and complementation mutants. **(A)** Design of the gene deletion target and sgRNA sequence for the *AfGsy2* gene using CRISPOR software. **(B)** Schematic representation of CRISPR-Cas9-mediated homologous recombination knockout of AfGsy2. **(C)** PCR verification of Δ*AfGsy2* mutants. Representative images from one of three independent biological replicates are shown. **(D)** Schematic representation of plasmid construction for *AfGsy2* overexpression and complementation strains. **(E)** Data are mean ± SD (n = 3 biological replicates), with individual biological replicate values overlaid as scatter points. Significance was determined by one-way ANOVA with Tukey’s post-hoc test; ****p < 0.0001; ns, not significant; n.d., not detected.

### 3.3 AfGsy2 regulates growth and stress tolerance of *A. flagrans*

To examine the role of AfGsy2 in growth, the WT and mutant strains were inoculated onto PDA, TG, and TYGA media and cultured for five days, with colony diameters measured daily. On PDA and TYGA, the Δ*AfGsy2* strains exhibited slower growth than the WT throughout the entire culture period. On TG medium, however, a growth defect in the Δ*AfGsy2* strains became evident only from day 3 onward (**Figures 3A, B**). In contrast, the OE*AfGsy2* and Δ*AfGsy2^C^* strains grew at rates comparable to the WT on all three media tested.

**Figure 3:**
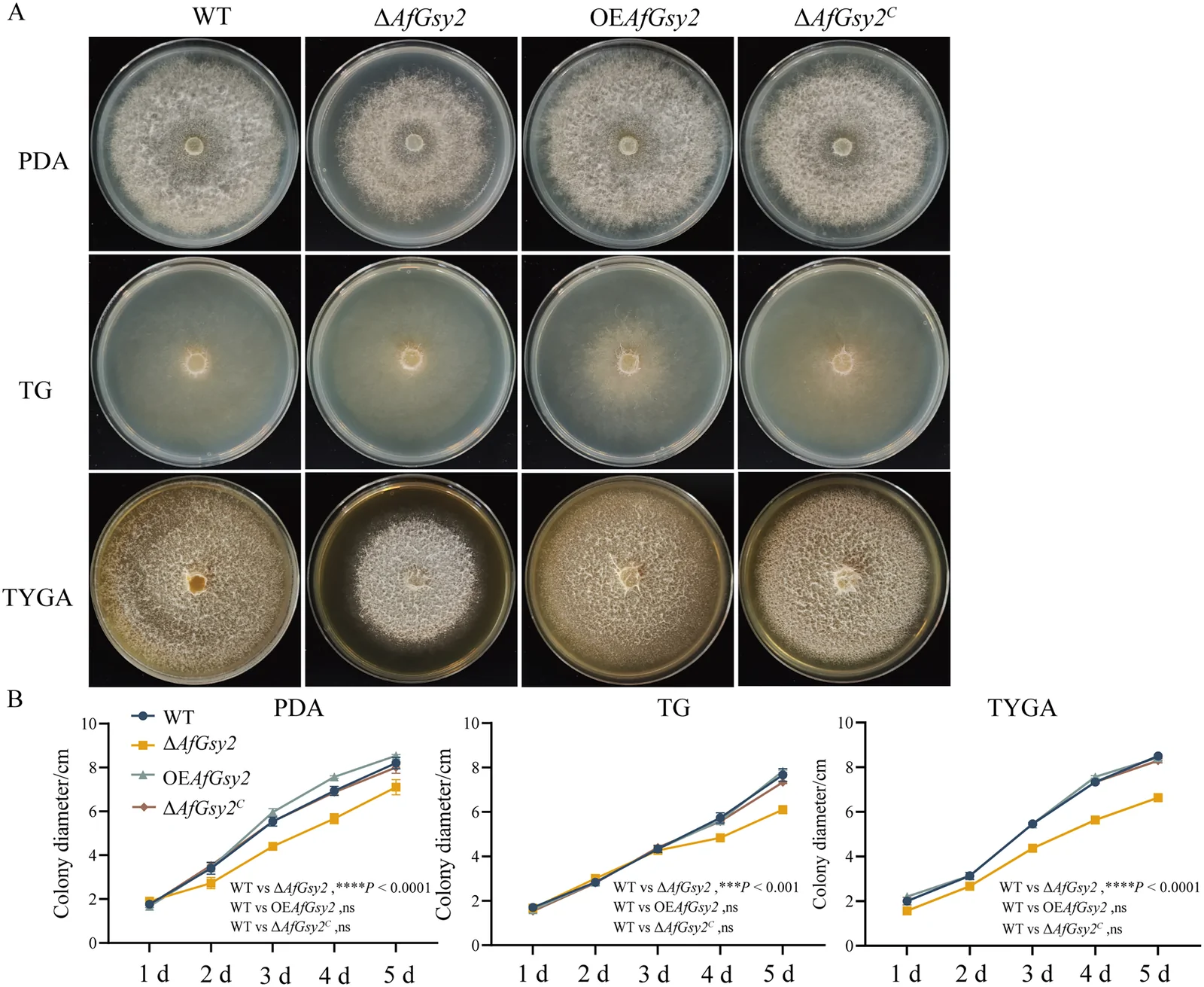
Growth of the wild-type (WT), Δ*AfGsy2*, OE*AfGsy2*, and Δ*AfGsy2^C^* strains on PDA, TG, and TYGA media. **(A)** Representative colony morphology after 5 days of cultivation. Representative images from one of three independent biological replicates are shown. **(B)** Quantification of mycelial growth rates. Data are mean ± SD (n = 3 biological replicates), with individual biological replicate values overlaid as scatter points. Significance was determined by two-way ANOVA with strain and time as factors, followed by Tukey’s post-hoc test. *p < 0.05, **p < 0.01, ***p < 0.001, ****p < 0.0001; ns, not significant.

To further assess the role of AfGsy2 in stress adaptation, the WT and mutant strains were grown on PDA supplemented with a gradient series of chemical stressors: sorbitol, Congo Red, SDS, NaCl, and H_2_O_2_. High concentrations of all five agents inhibited the growth of all strains tested (**Figure 4**; **Supplementary Figure 1**). Compared with the WT, the Δ*AfGsy2* strains exhibited reduced sensitivity to sorbitol (0.25, 0.5, and 1 M), NaCl (0.1 M), and H_2_O_2_ (2.5 mM). Notably, a low concentration of Congo Red (25 μg/mL) partially rescued the growth defect of the Δ*AfGsy2* strains, whereas at higher concentrations (50 and 100 μg/mL), Congo Red inhibited growth, although the mutant strains remained less sensitive than the WT strain. The complemented strain (Δ*AfGsy2^C^*) and the overexpression strain (OE*AfGsy2*) displayed stress tolerance phenotypes comparable to those of the WT. (**Figure 4**; **Supplementary Figure 1**).

**Figure 4:**
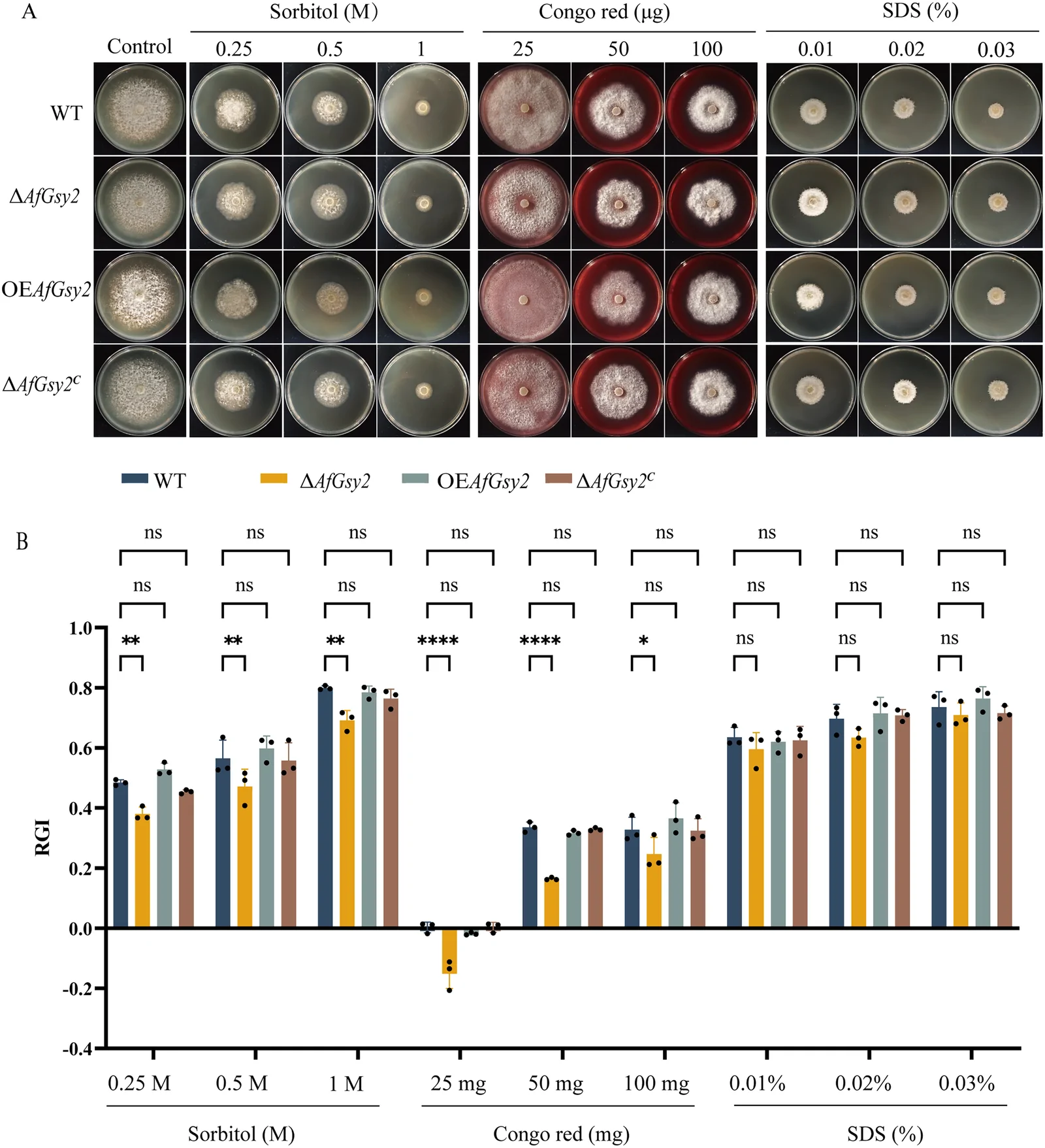
Comparison of stress response to chemical agents between the WT and mutant strains. **(A)** Colony morphology of the WT and mutant strains (Δ*AfGsy2*, OE*AfGsy2*, and Δ*AfGsy2^C^*) cultured on PDA medium supplemented with different chemical agents. Representative images from one of three independent biological replicates are shown. **(B)** Relative growth inhibition (RGI) values of the WT and mutant strains on media supplemented with different chemical agents. Data are mean ± SD (n = 3 biological replicates), with individual biological replicate values overlaid as scatter points. Significance was determined by two-way ANOVA with strain and concentration as factors, followed by Tukey’s post-hoc test. *p < 0.05, **p < 0.01, ***p < 0.001, ****p < 0.0001.

### 3.4 AfGsy2 positively regulates trap formation and nematicidal activity

To define the role of AfGsy2 in trap morphogenesis and predatory activity, we exposed WT and mutant strains to *C. elegans* and quantified trap numbers and nematode mortality at 12, 24, and 36 h. Deletion of *AfGsy2* significantly delayed trap formation and reduced nematicidal activity, whereas overexpression of *AfGsy2* accelerated both processes (**Figure 5**). At 36 h, the Δ*AfGsy2* strains produced 259.0 traps/cm², substantially fewer than the WT (369.0 traps/cm²) (**Figures 5A, C**), and achieved a nematode mortality of 72.5%, compared with 94.9% for the WT (**Figures 5B, D**). In contrast, the OE*AfGsy2* strains generated 678.0 traps/cm² at 36 h (**Figures 5A, C**), exceeding the WT, and resulted in a mortality rate of 100% (**Figures 5B, D**). The complemented strain Δ*AfGsy2^C^* produced 362.0 traps/cm² and caused 92.8% mortality, restoring both parameters to levels comparable to those of the WT. These results indicate that AfGsy2 functions as a positive regulator of trap formation and predation efficiency, suggesting its potential relevance to the predatory activity of *A. flagrans*.

**Figure 5:**
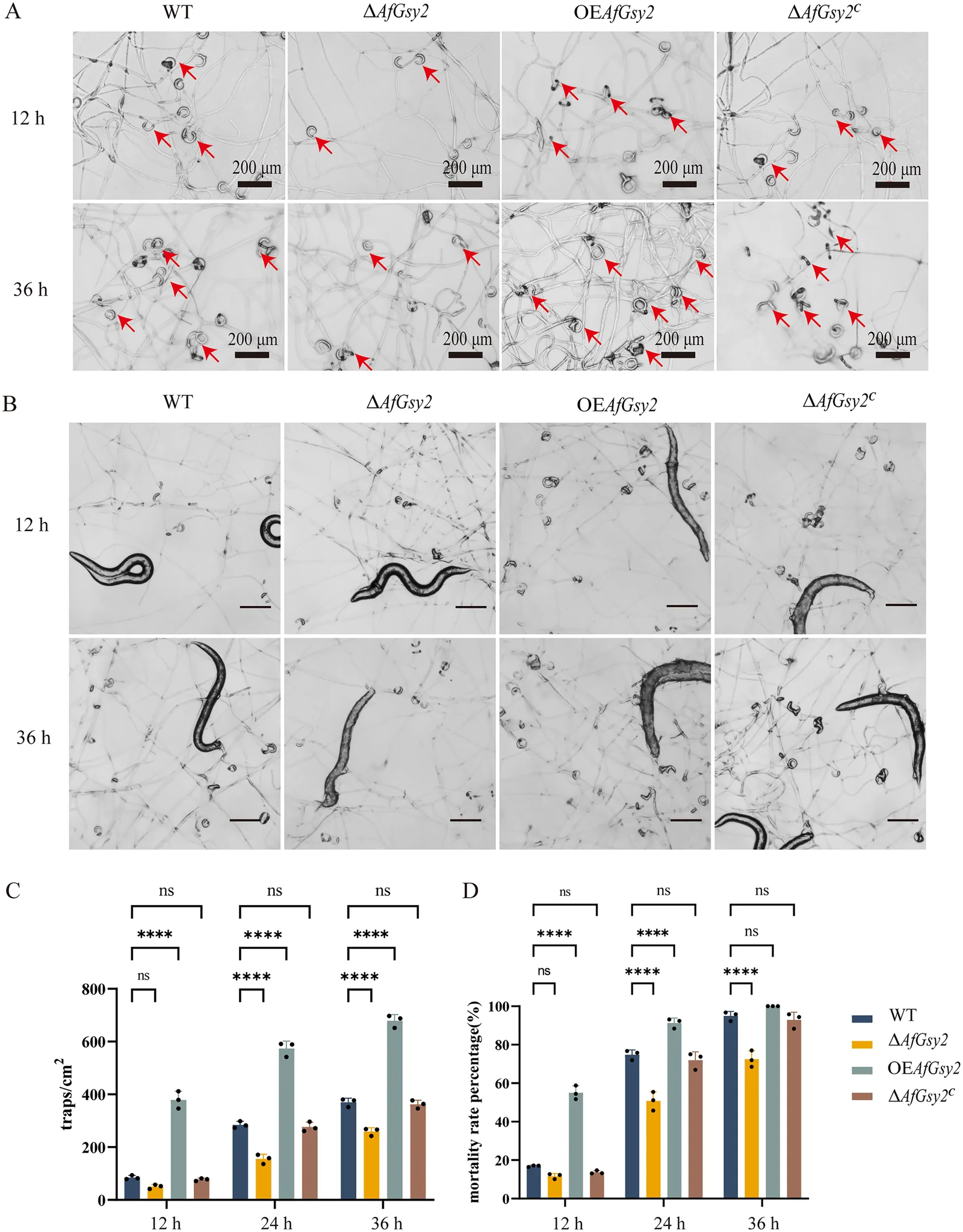
Trap formation and nematicidal activity of the WT and mutant strains (Δ*AfGsy2*, OE*AfGsy2*, and Δ*AfGsy2^C^*). **(A)** Representative trap morphology at 12 and 36 h. Scale bar, 200 μm. Red arrowheads indicate traps. **(B)** Nematode capture and degradation by the indicated strains at 12 and 36 h. Scale bar, 100 μm. Representative images from one of three independent biological replicates are shown. **(C)** Quantification of trap numbers **(D)** nematode mortality rates at the indicated time points. Data are mean ± SD (n = 3 biological replicates), with individual biological replicate values overlaid as scatter points. Significance was determined by two-way ANOVA with strain and time as factors, followed by Tukey’s post-hoc test. *p < 0.05, **p < 0.01, ***p < 0.001, ****p < 0.0001.

### 3.5 AfGsy2 regulates chlamydospore production, glycogen accumulation, and germination in *A. flagrans*

To investigate the role of AfGsy2 in chlamydospore formation, the WT and the mutant strains were inoculated onto 6 cm WA plates and cultured for 14 days. Chlamydospore production was then examined microscopically. The Δ*AfGsy2* mutant strains generated morphologically normal chlamydospores but in significantly reduced numbers (506.0 spores/cm^2^, approximately 68.8% of the WT level) (**Figures 6A, B**). In contrast, the OE*AfGsy2* strains yielded 812.0 spores/cm^2^, approximately 1.10-fold that of the WT strain, whereas the Δ*AfGsy2^C^* strain restored chlamydospore production to 709.0 spores/cm^2^, equivalent to the WT level (735.0 spores/cm^2^) (**Figure 6B**). Thus, AfGsy2 functions as a key positive regulator of chlamydospore formation.

**Figure 6:**
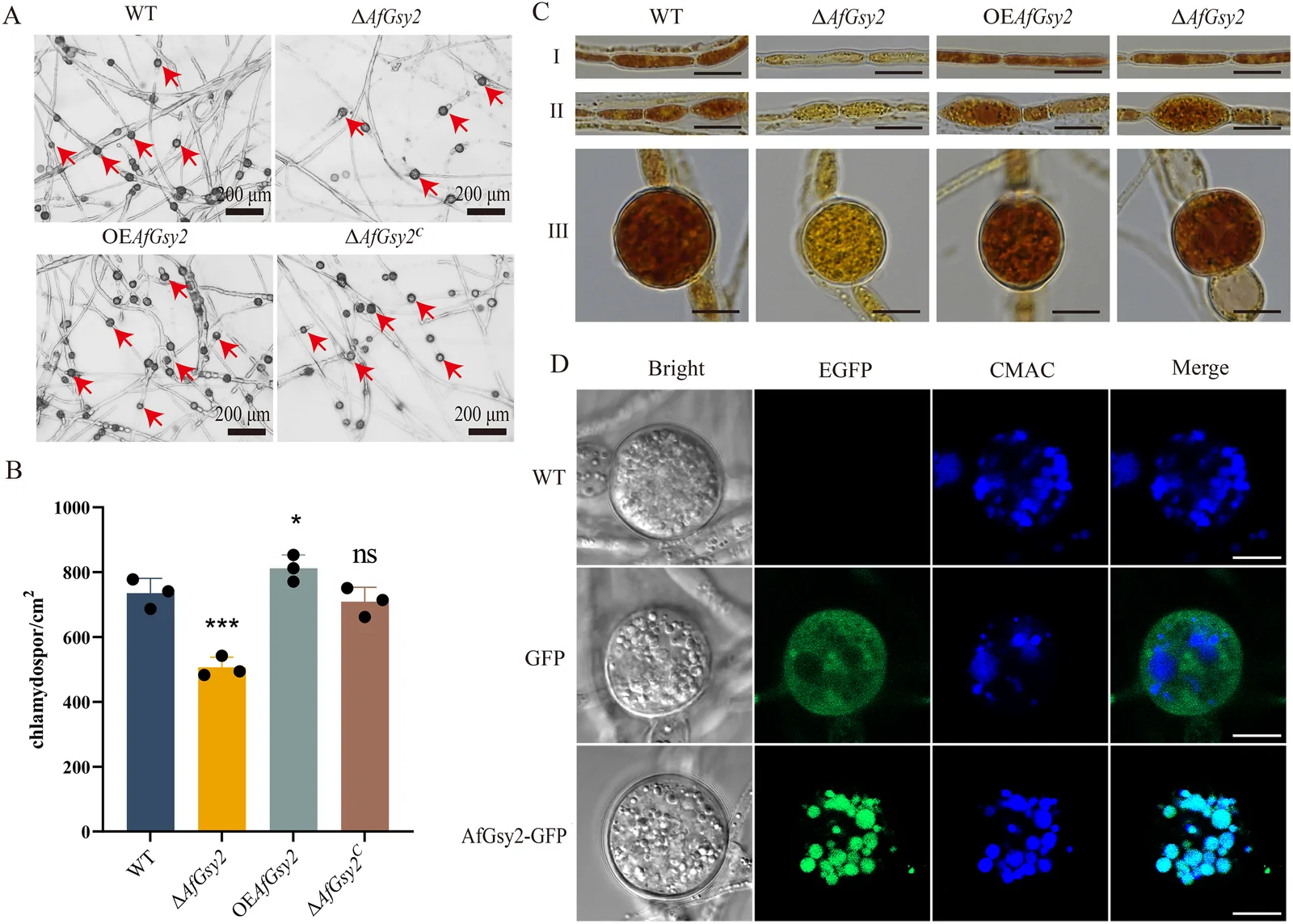
Functional analysis of AfGsy2 in chlamydospore formation and glycogen accumulation. **(A)** Morphology of chlamydospores produced by the WT and mutant strains (Δ*AfGsy2*, OE*AfGsy2*, Δ*AfGsy2^C^*) after 14 days on WA medium. Scale bar, 200 μm. Red arrowheads indicate chlamydospores. **(B)** Quantification of chlamydospore numbers. Data are mean ± SD (n = 3 biological replicates), with individual biological replicate values overlaid as scatter points. Significance was determined by one-way ANOVA with Tukey’s post-hoc test. *p < 0.05, **p < 0.01, ***p < 0.001, ****p < 0.0001. **(C)** Glycogen staining at different developmental stages: I, hyphal stage; II, middle stage; III, chlamydospore stage. Scale bar, 10 μm. **(D)** Subcellular localization of AfGsy2-GFP in chlamydospore. Scale bar, 10 μm. Representative images from one of three independent biological replicates are shown.

To assess the role of AfGsy2 in glycogen accumulation, Lugol’s iodine staining was performed on hyphae, swelling hyphae, and mature chlamydospores. During chlamydospore development, the intensity of Lugol’s iodine staining increased progressively as hyphae swelled (**Figure 6C**). Notably, the Δ*AfGsy2* mutant strains exhibited reduced staining intensity compared with the WT strain, indicating that *AfGsy2* deficiency results in reduced glycogen accumulation in both hyphae and chlamydospores. In contrast, the OE*AfGsy2* and Δ*AfGsy2^C^* strains displayed staining intensities similar to that of the WT strain. Collectively, these visualization results of glycogen staining indicate that the expression level of AfGsy2 is positively correlated with intracellular glycogen accumulatino. Furthermore, we expressed an AfGsy2-GFP fusion protein under the control of the *AfH2B* promoter. Phenotypic observation confirmed that expression of the AfGsy2-GFP fusion protein did not compromise the biological functions of *A. flagrans* (**Supplementary Figure 2**), while serving as an effective marker for subcellular localization. Combined with CMAC staining solution, confocal microscopy revealed that AfGsy2-GFP predominantly localized to the vacuoles of chlamydospores (**Figure 6D**).

We further examined the effect of AfGsy2 on chlamydospore germination. Compared to the WT strain, the Δ*AfGsy2* mutant strains exhibited significantly slower germ tube elongation and markedly reduced germination rates during the first 12 h of the assay (3 h: 3.5% vs. 7.7%; 6 h: 9.8% vs. 21.9%; 12 h: 55.3% vs. 78.8%) (**Figure 7**). In the OE*AfGsy2* strains, germination proceeded at rates similar to those of the WT (3 h: 8.1%; 6 h: 20.7%; 12 h: 77.3%) (**Figure 7**), and the complemented strains Δ*AfGsy2^C^* also restored germination to WT levels. Together, these findings establish AfGsy2 as a positive regulator of chlamydospore germination.

**Figure 7:**
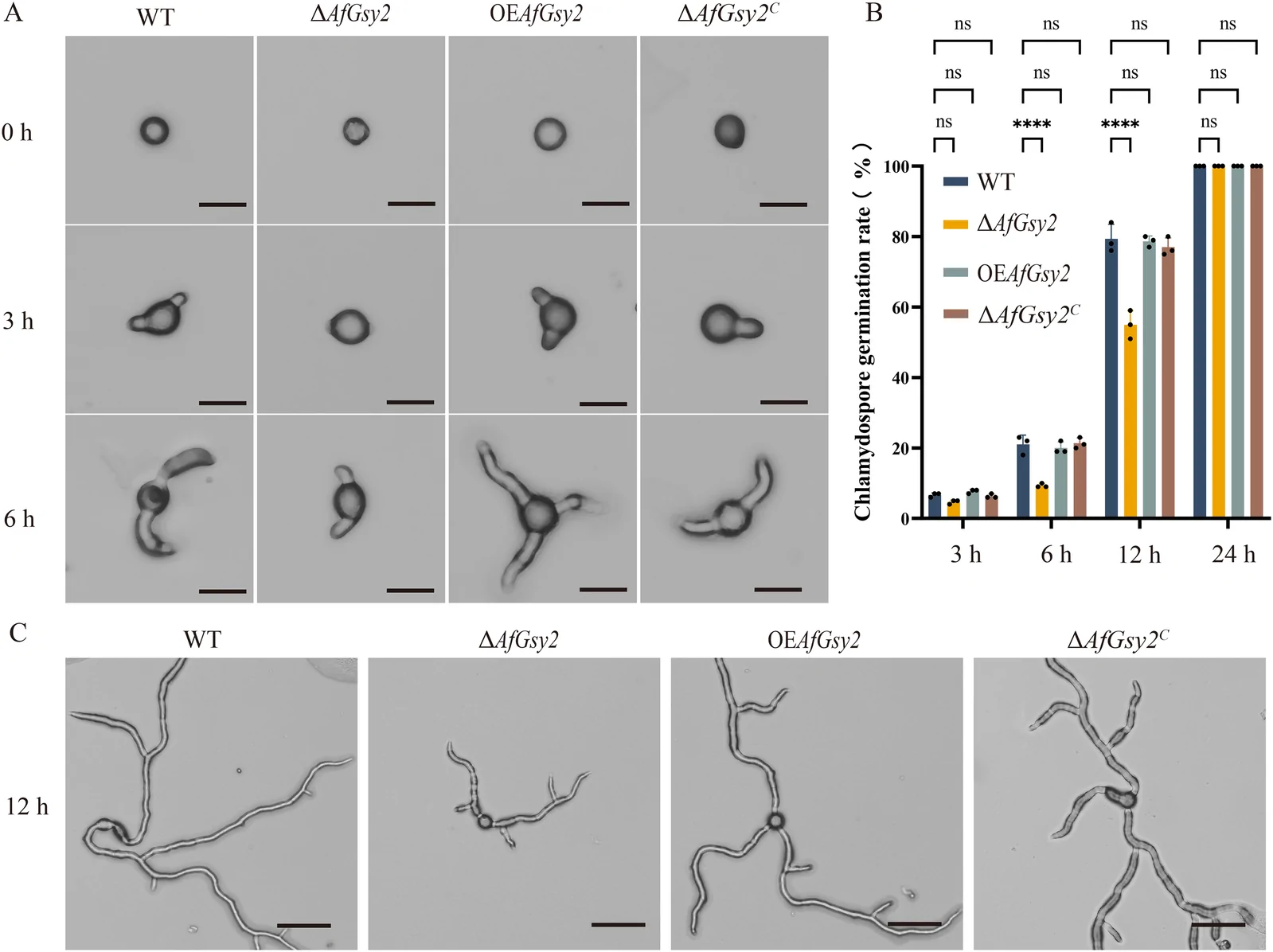
Effect of AfGsy2 on chlamydospore germination. **(A)** Germination status of chlamydospores at 3, 6, and 12 h. Scale bar, 25 μm. **(B)** Quantification of germination rates at 3, 6, 12, and 24 h. Data are mean ± SD (n = 3 biological replicates), with individual biological replicate values overlaid as scatter points. Significance was determined by two-way ANOVA with strain and time as factors, followed by Tukey’s *post-hoc* test. *p < 0.05, **p < 0.01, ***p < 0.001, ****p < 0.0001. **(C)** Representative images of germinating chlamydospores at 12 h. Scale bar, 50 μm.

## 4 Discussion

Glycogen synthase (GSY) is the key enzyme catalyzing glycogen biosynthesis and is highly conserved across eukaryotes (**Supplementary Figure 3**; Loza and Doering, 2024). However, its functional roles in fungi occupying specialized ecological niches, particularly nematode-trapping fungi, remain poorly defined. In this study, we provide the first systematic functional characterization of AfGsy2 in the nematode-trapping fungus *A. flagrans*. Using *C. elegans* as a model, our findings indicate that AfGsy2 serves not only as the key enzyme for glycogen synthesis but also plays a pleiotropic role in regulating vegetative growth, stress adaptation, chlamydospore formation, and predatory activity in *A. flagrans*. By directly coupling glycogen metabolism to fungal survival, adaptation, and predatory activity, this work identifies a tractable molecular target for improving nematode-trapping fungi.

The regulation of growth and stress tolerance by AfGsy2 involves a complex mechanism extending beyond mere energy supply. Our experimental data demonstrate that the Δ*AfGsy2* mutant strains exhibited retarded growth on both nutrient-rich (PDA, TYGA) and nutrient-poor (TG) media, supporting the importance of glycogen as a basal carbon and energy reserve for sustaining hyphal polarized growth. Similar growth defects upon disruption of glycogen metabolism have been reported in *C. albicans* and *Aspergillus nidulans* (Flipphi et al., 2009; Miao et al., 2024). More strikingly, the Δ*AfGsy2* mutant strains displayed enhanced tolerance to sorbitol (osmotic stress), NaCl (ionic stress), and H_2_O_2_ (oxidative stress), whereas low concentrations of Congo red partially rescued their growth defect. These results indicate that loss of AfGsy2 does not simply reduce general fitness but alters stress-responsive programs. However, the mechanisms underlying these phenotypes remain to be determined.

AfGsy2 positively regulates predatory activity. Deletion of *AfGsy2* substantially impaired trap formation and nematode-killing activity, whereas its overexpression resulted in faster and more efficient predation. These results suggest a link between glycogen metabolism and the formation of the adhesive three-dimensional networks, a highly specialized and energy-demanding predatory structure. Trap morphogenesis involves extensive cytoskeletal reorganization, cell wall remodeling, and protein synthesis and secretion (Yang et al., 2007; Liang et al., 2019), processes that require substantial energy and carbon skeletons. We therefore propose that glycogen synthesized by AfGsy2 serves as a critical energy reservoir for trap formation, conceptually paralleling the dependence of *Magnaporthe oryzae* appressorium development on glycogen mobilization (Badaruddin et al., 2013; Zhang et al., 2023a). However, unlike *M. oryzae*, where blocking glycogen synthesis alone does not significantly affect pathogenicity, deletion of the synthase AfGsy2 alone severely attenuated predatory activity in *A. flagrans*. Likewise, the delayed germination of Δ*AfGsy2* chlamydospores further underscores that glycogen storage, rather than its immediate mobilization, is critical for developmental programs that require pre-accumulated energy (**Figure 7**). Insufficient glycogen reserves likely delay both trap formation and spore germination, thereby impairing rapid host capture and subsequent propagation.

AfGsy2 also controls chlamydospore development. In *A. flagrans*, Δ*AfGsy2* mutants produced morphologically normal chlamydospores but in significantly reduced numbers, whereas OE*AfGsy2* produced more chlamydospores and the complemented strain restored production to WT levels. Glycogen, as the major cytosolic carbon storage polysaccharide in fungi, plays a central role in spore development and germination. In *M. oryzae*, deletion of *gsn1* (glycogen synthase) abolishes glycogen deposition throughout conidia and appressorium development, confirming glycogen synthase as essential for glycogen accumulation (Badaruddin et al., 2013). In *A. flagrans*, Lugol’s iodine staining intensity suggested that AfGsy2 positively correlates with both chlamydospore yield and intracellular glycogen content. Because chlamydospores are the active ingredient of biocontrol agents, these traits directly influence shelf life and field efficacy (Jiang et al., 2017). Although per-spore glycogen staining in OE*AfGsy2* was not significantly higher than WT, total chlamydospore production increased markedly, suggesting that AfGsy2 overexpression may accelerate energy allocation toward sporulation. Furthermore, we observed that AfGsy2-GFP predominantly localized to the vacuoles of chlamydospores (**Figure 6D**), which differs from the glycogen-dependent distribution pattern of Gsy2p in *S. cerevisiae*, where Gsy2p is diffuse in the cytoplasm at high glycogen, punctate at low glycogen, and nuclear in the absence of glycogen (Wilson et al., 2010). The vacuolar localization of AfGsy2-GFP may reflect its participation in glycogen turnover within the vacuole.

Working hypotheses. Despite these findings, the mechanistic basis remains largely unknown. We propose the following non-mutually exclusive hypotheses. First, loss of AfGsy2 may redirect carbon flux toward trehalose or glycerol synthesis, since glycogen and trehalose synthesis compete for UDP-glucose (François and Parrou, 2001), and trehalose stabilizes proteins and membranes (Fillinger et al., 2001; Elbein et al., 2003). This could explain the enhanced stress tolerance of Δ*AfGsy2*, but trehalose and glycerol levels were not measured. Second, altered glycogen metabolism may be linked to cell wall integrity (CWI) signaling. The partial rescue by low Congo red and altered sensitivity to cell wall stressors raise this possibility. In *C. neoformans*, glycogen organization is coupled to cell wall β-1,3-glucan via the glucan-organizing enzyme Goe1 (Loza and Doering, 2024), and the CWI pathway senses cell wall stress (Levin, 2011). However, whether AfGsy2 affects CWI signaling in *A. flagrans* is unknown. Third, AfGsy2 activity may be post-translationally regulated. In *S. cerevisiae*, phosphorylation of Gsy2p inhibits enzyme activity (Wilson et al., 2010), and GSK3-family kinases regulate glycogen metabolism in *Ganoderma lucidum* (Miao et al., 2024; Wang et al., 2025). Whether such regulation occurs in *A. flagrans* remains unknown. Finally, STRING and AlphaFold3 analyses identified 10 candidate AfGsy2-associated proteins, including core glycogen metabolic enzymes, phosphoglucomutase, UTP–glucose-1-phosphate uridylyltransferase, and trehalose biosynthesis components, all of which were significantly upregulated during chlamydospore formation (**Supplementary Figures 4–6**). These are purely computational predictions and do not provide experimental evidence for physical interactions; the ipTM values were generally below the threshold for stable binary complexes. Co-immunoprecipitation, yeast two-hybrid, or proximity labeling will be required for validation.

Several limitations should be acknowledged. All predatory activity assays were performed using *C. elegans* as a laboratory model rather than plant-parasitic nematodes. While *C. elegans* provides a well-standardized and genetically tractable system for quantifying trap morphogenesis and basal nematocidal activity, it does not fully recapitulate the complexity of interactions with plant-parasitic nematodes in soil or plant environments. Future work should quantify trehalose and glycerol, analyze cell wall composition, extend assays to plant-parasitic nematodes in soil or plant systems, and dissect the molecular mechanism of AfGsy2, including the identification and experimental validation of its interacting proteins by co-immunoprecipitation, yeast two-hybrid, or proximity labeling, as well as functional characterization of these interactions during chlamydospore development and trap formation.

In summary, this functional characterization of AfGsy2 reveals a previously unexplored link between glycogen metabolism and growth, stress tolerance, sporulation, and predatory activity in a nematode-trapping fungus. These observations contribute to a better understanding of the physiological and ecological strategies of predatory fungi and provide a molecular basis for future investigations into the biocontrol potential of *A. flagrans*.

## Data Availability

The datasets presented in this study can be found in online repositories. The names of the repository/repositories and accession number(s) can be found in the article/**Supplementary Material**.
