# Supplementary material for: Glycogen synthase AfGsy2 regulates mycelial growth, stress response, predatory activity, and chlamydospore development in the nematode-trapping fungus Arthrobotrys flagrans
Source: Front Cell Infect Microbiol. 2026 Oct 7;16:1891167. doi: 10.3389/fcimb.2026.1891167 (PMC13683002; doi:10.3389/fcimb.2026.1891167)
Supplement: Supplementary file 1 [file DataSheet1.docx]

Supplementary Material

Glycogen synthase AfGsy2 regulates mycelial growth, stress response, predatory activity, and chlamydospore development in the nematode-trapping fungus *Arthrobotrys flagrans*

Qianfei Shi ^1†^, Yu Zhang ^1,2†^, Minghe Mo ^1*^, Guohong Li ^1*^

^1^State key Laboratory for Conservation and Utilization of Bio-Resources in Yunnan, Yunnan Key Laboratory of Basic Research and Innovative Application for Green Biological Production, Yunnan University, Kunming 650500, China.

^2^School of Ecology and Environment, Yunnan University, Kunming 650500, China.

^†^These authors contributed equally to this article.

*** Correspondence:**

Guohong Li: ligh@ynu.edu.cn

Minghe Mo: minghemo@163.com

# 1 Supplementary Figures

**Supplementary Figure 1.** Comparison of stress response to chemical agents between the WT and mutant strains (Δ*AfGsy2*, OE*AfGsy2*, andΔ*AfGsy2^C^*). (A) Colony morphology of the WT and mutant strains cultured on PDA medium supplemented with NaCl and H_2_O_2_. (B) Relative growth inhibition (RGI) values of the WT and mutant strains on media supplemented with different chemical agents. Data are mean ± SD (n = 3 biological replicates), with individual biological replicate values overlaid as scatter points. Significance was determined by two‑way ANOVA with strain and concentration as factors, followed by Tukey’s post‑hoc test. **p* < 0.05, ***p* < 0.01, ****p* < 0.001, *****P* < 0.0001).

Supplementary Figure 2. Phenotypic comparison of wild‑type (WT), non‑tagged overexpression (n‑AfGsy2), and GFP‑tagged overexpression (AfGsy2‑GFP) strains. (A) Comparison of mycelial growth rates among WT, n‑AfGsy2, and AfGsy2‑GFP strains on three different media. (B) Quantification of trap numbers at the indicated time points (12 h, 24 h, and 36 h). (C) Nematode mortality rates at the indicated time points. (D) Chlamydospore yields of the three strains. Data are presented as mean ± SD (n = 3 biological replicates), with individual replicate values overlaid as scatter points. Significance was determined by two‑way ANOVA with strain and time as factors for (A–C), and by one‑way ANOVA with strain as the factor for (D), followed by Tukey's post‑hoc test. **p* < 0.05, ***p* < 0.01, ****p* < 0.001, *****p* < 0.0001; ns, not significant. Both n‑AfGsy2 and AfGsy2‑GFP strains exhibited consistently enhanced phenotypes across all measured parameters compared with the WT strain, confirming that the GFP tag does not compromise AfGsy2 function.

**Supplementary Figure 3.** Schematic diagram of glycogen metabolism in fungi. The pathway comprises priming (glycogenin, Glg, catalyzing autoglucosylation to generate a short oligosaccharide primer), chain extension (glycogen synthase, Gsy2), branching (glycogen branching enzyme, Glc), and degradation (glycogen phosphorylase, Gph; glycogen debranching enzyme, Gdb). Glycogen synthase (Gsy2) catalyzes the rate‑limiting step of the pathway and is the central focus of this study (AfGsy2 in *A. flagrans*). This diagram is based on the conserved glycogen metabolic pathway documented in fungal model systems (Loza and Doering, 2024).

**Supplementary Figure 4.** Prediction and expression profiling of AfGsy2-interacting proteins. (A) Protein–protein interaction network of AfGsy2 in *A. flagrans*, generated using the STRING database (v12.0) with a high-confidence interaction score threshold (≥0.700). Nodes represent the ten candidate proteins: DFL_008525 (1,4-α-glucan-branching enzyme), DFL_000800 (α-1,4 glucan phosphorylase), DFL_008491 (glycogenin glucosyltransferase), DFL_008885 (glycogen debranching enzyme), DFL_004634 (phosphoglucomutase), DFL_005058 (UTP–glucose-1-phosphate uridylyltransferase), DFL_003363 (trehalose-6-phosphate synthase), DFL_000338 (trehalose-phosphatase), DFL_004044 (CBM21 domain-containing protein phosphatase), and DFL_008492 (predicted glycosyltransferase). (B) Transcriptomic analysis of the eleven predicted partners during chlamydospore development, including the ten proteins listed in (A) plus GSK3 (DFL_007711), a glycogen synthase kinase known to phosphorylate glycogen synthase in other fungi. (C) AlphaFold3-based structural assessment of AfGsy2–candidate binary complexes. Global model confidence was assessed by the predicted template modeling (pTM) score, and interface confidence was quantified by the interface predicted template modeling (ipTM) score. Following established guidelines, ipTM > 0.75 was considered indicative of a highly probable direct interaction, 0.5–0.75 of moderate confidence, and < 0.5 of a low likelihood for a stable physical complex.

**Supplementary Figure 5.** AlphaFold 3-predicted complex structures of Gsy with candidate proteins, colored by confidence. (A–K) Predicted the structures of Gsy with other proteins. Models are colored by per-residue pLDDT scores: blue (very high, >90), cyan (confident, 90–70), yellow (low, 70–50), and orange (very low, <50). The ipTM and pTM scores are indicated for each prediction.

**Supplementary Figure 6.** AlphaFold 3-predicted complex structures of Gsy with candidate proteins, colored by chains. (A–K) Predicted the structures of Gsy (red) with other proteins (yellow). The ipTM and pTM scores are indicated above each model.

# 2 Supplementary Tables

**Supplementary table 1.** *A. flagrans* strains and plasmids used in this study.

| **Strains/Plasmids** | **Genotype** | **Description** |
| --- | --- | --- |
| *A. flagrans* | WT | WT |
| Δ*AfGsy2* | Δ*AfGsy2*; *hph* | Gene deletion of *AfGsy2* |
| OE*AfGsy2* | *H2B(p)*:: *AfGsy*::*GFP*::*H2B(t)*;*G418* | Overexpression of *AfGsy2* |
| Δ*AfGsy2^C^* | Δ*AfGsy2*; *G418*  *AfGsy2(p)*:: *AfGsy2*::*AfGsy2(t)*;*G418* | Complementation of *AfGsy2* |

**Supplementary table 2.** Primers used in this study.

| **Primers** | **Primer sequences (5'-3')** | | **Description** |
| --- | --- | --- | --- |
| *Gpd-*for | CGAGAAGCCCGCCAAGT | | qPCR |
| *Gpd*-rev | AAAGGTGTCGGTCAAAGCAAT | |  |
| RT-*AfGsy2-*for | AGAGGAGTTGGCTGGCTGTAC | |  |
| RT-*AfGsy2*-rev | TTGATGGCTGCGGTGAGG | |  |
| Δ*AfGsy2*-up*-*for | ttcggatcttccagaACAGATAGACCCTTGAGGCACCG | Amplify the 5' homologous fragment of *AfGsy2* | |
| Δ*AfGsy2*-up-rev | tcatcttctgTGATTGGCGCTTTCGACTTG |  |  |
| Δ*AfGsy2*-*hyg-*for | gcgccaatcaCAGAAGATGATATTGAAGGAGCATTT | Amplify the *hph* cassette | |
| Δ*AfGsy2*-*hyg*-rev | atctggcgccaAAAGAAGGATTACCTCTAAACAAGTGTACC |  |  |
| Δ*AfGsy2-*down-for | tccttctttTGGCGCCAGATACAACCTG | Amplify the 5' homologous fragment of *AfGsy2* | |
| Δ*AfGsy2*-down*-*rev | caactgccgttcgacCCCTGTTCGATGACATGGACG |  |  |
| OE*AfGsy2*-*KU70*-up*-*for | aaaacgacggccagtAGTTTGAGGATAAGACGGAGCCG | Amplify the *ku70*-up cassette | |
| OE*AfGsy2*-*KU70*-up*-*rev | ttgctcctttcAGAACGGACCCACCCACG |  |  |
| OE*AfGsy2*-*H2B(P)*-for | gtccgttctGAAAGGAGCAAAATCGAGGGA | Amplify the *H2B(****Promoter****)* cassette | |
| OE*AfGsy2*-*H2B(P)*-rev | gtttaccgtcgcccatTTTGAAATTTGTTTTTTGTTTGGG |  |  |
| OE*AfGsy2-*for | caaaATGGGCGACGGTAAACGAG | Amplify the *AfGsy2* cassette | |
| OE*AfGsy2-*rev | ttgctcacGTTCCCGTTGGTGCCAGC |  |  |
| OE*AfGsy2*-*EGFP*-*H2B(T)*-for | accaacgggaacGTGAGCAAGGGCGAGGAGC | Amplify the *EGFP*-*H2B(****Terminator****)* cassette | |
| OE*AfGsy2*-*EGFP*-*H2B(T)*-rev | caatcatatcgGTCAGATTGAATCTGAAATAAAGGGA |  |  |
| OE*AfGsy2*-*KU70*-down-G418-for | caatctgacCGATATGATTGAACAAGATGGATTG | Amplify the *ku70*-down-G418 cassette | |
| OE*AfGsy2*-*KU70*-down-G418-rev | ccgggtaccgagctcgaattcCTACCGAATCCCTAATGGCGA |  |  |
| Δ*AfGsy2^C^-*KU70-up*-*for | gggtgggtccgttctGCGGCCGCATGGTGAGCAAGGGCGAGGAG | Amplify the *ku70*-up cassette | |
| Δ*AfGsy2^C^*-KU70-up*-*rev | aTTACTTGTACAGCTCGTCCATGCC |  |  |
| Δ*AfGsy2^C^-KU70*-down-G418*-*for | ggacgagctgtacaagtaaTACTAATCAGCTGGACTAGTCGTTTTT | Amplify the *ku70*-down-G418 cassette | |
| Δ*AfGsy2^C^-KU70*-down-G418*-*rev | caatatcatcttctgGCTTTAAGAAGAACAAGTAGTAATAGTAGTAGTAGT |  |  |
| Δ*AfGsy2^C^-*EGFP-*AfGsy*(T)-G418*-*for | gggtgggtccgttctAATTCTTGGCCATTGATCGTTC | Amplify the *EGFP*-*AfGsy(****Terminator****)* cassette | |
| Δ*AfGsy2^C^-EGFP*-*AfGsy(T)*-G418*-*rev | gcccttgctcaccatATCCAGTCTAGCGATCATCTCGTC |  |  |
| Δ*AfGsy2^C^-AfGsy2*(P)-*AfGsy-*for | gggtgggtccgttctGGAAAAATATCACAAACTTTTTGACG | Amplify the *AfGsy(****Promoter****)*-*AfGsy* cassette | |
| Δ*AfGsy2^C^-AfGsy2*(P)-*AfGsy2-*rev | gcccttgctcaccatCTAGTTCCCGTTGGTGCCAG |  |  |
| F*-*for | CGAACTGAACTCCACCAAACAA | **Screening and verification of Δ*AfGsy2* strains** | |
| R*-*rev | CGACATACCTCTCCCAAAAACC |  |  |

**Supplementary table 3.** Protein details in the protein-protein interaction network of AfGsy2.

| **Gene ID** | **Descirption** | **score** |
| --- | --- | --- |
| DFL_008525 | 1,4-alpha-glucan-branching enzyme | 0.994 |
| DFL_004044 | CBM21 domain-containing protein; protein phosphatase | 0.987 |
| DFL_000800 | Alpha-1,4 glucan phosphorylase | 0.975 |
| DFL_008492 | Uncharacterized protein; transferase activity, transferring glycosyl groups | 0.972 |
| DFL_008491 | glycogenin glucosyltransferase | 0.972 |
| DFL_000338 | Trehalose-phosphatase | 0.946 |
| DFL_004634 | Phosphoglucomutase | 0.937 |
| DFL_005058 | UTP-glucose-1-phosphate uridylyltransferase | 0.918 |
| DFL_008885 | Glycogen debranching enzyme | 0.914 |
| DFL_003363 | Trehalose-6-phosphate synthase | 0.907 |
| DFL_007711 | Glycogen synthase kinase GSK3 | — |

Note: "—" indicates that DFL_007711 was not detected by STRING database, hence no score was assigned.
